# The Syk/CARD9-coupled receptor Dectin-1 is not required for host resistance to *Mycobacterium tuberculosis* in mice

**DOI:** 10.1016/j.micinf.2010.10.013

**Published:** 2011-02

**Authors:** Mohlopheni J. Marakalala, Reto Guler, Lungile Matika, Graeme Murray, Muazzam Jacobs, Frank Brombacher, Antonio Gigliotti Rothfuchs, Alan Sher, Gordon D. Brown

**Affiliations:** a Division of Immunology, Institute of Infectious Diseases and Molecular Medicine, University of Cape Town, South Africa; b International Centre for Genetic Engineering and Biotechnology, Cape Town, South Africa; c Section of Translational Medicine, Division of Applied Medicine, University of Aberdeen, UK; d Laboratory of Parasitic Diseases, National Institute of Allergy and Infectious Diseases, USA; e Department of Microbiology, Tumor and Cell Biology, Karolinska Institute, Sweden; f Section of Immunology and Infection, Division of Applied Medicine, Institute of Medical Sciences, Foresterhill, University of Aberdeen, Aberdeen AB25 2ZD, UK

**Keywords:** C-type lectin receptors, Syk, CARD9, Dectin-1, Pulmonary disease, Inflammation

## Abstract

There is interest in identifying the pattern recognition receptors involved in initiating protective or non-protective host responses to *Mycobacterium tuberculosis* (Mtb). Here we explored the role of the Syk/CARD9-coupled receptor, Dectin-1, using an aerosol model of Mtb infection in wild-type and Dectin-1 deficient mice. We observed a reduction in pulmonary bacilli burdens in the Dectin-1 deficient animals, but this did not correlate with significant changes in pulmonary pathology, cytokine levels or ability of these animals to survive the infection. Thus Dectin-1 makes a minor contribution to susceptibility to Mtb infections in mice.

Of all the human diseases resulting from bacterial infection, tuberculosis (TB) remains the deadliest, killing about 1.6 million people a year globally [1]. Host control of Mtb relies heavily on the activation of interferon-γ (IFN-γ)-producing CD4^+^ T cells, and cytokines involved in the development of T_H_1 responses, such as IL-12, are critical for the control of infections with this pathogen [2]. Conversely, “suppressive” cytokines such as IL-10 negatively regulate macrophage function during mycobacterial infection, although they may be required for limiting inflammation associated pathology [3].

The initiation of these responses in the naïve host relies on pattern recognition receptors (PRRs), including several Toll-like (TLRs) and C-type lectin receptors (CLR) [4]. Of particular interest are the CLRs which induce intracellular signalling through the Syk/CARD9 pathway, which was recently shown to be essential for the control of Mtb [5]. Two CLRs which signal via this pathway have been shown to recognise mycobacteria, and include Mincle, which binds mycobacterial cord factor [6,7], and Dectin-1, whose mycobacterial ligand is unknown [8].

Dectin-1 is traditionally associated with the recognition of fungi, but this receptor has also been implicated in anti-mycobacterial immunity. Dectin-1 is highly expressed on alveolar macrophages [8], and can be induced by Mtb on airway epithelial cells [9], and *in vitro* studies have demonstrated a contribution of this receptor in mycobacterial uptake, induction of the respiratory burst, and the production of cytokines, including IL-6, IL-12 and IL-17 [10–13]. To explore the role of Dectin-1 in the host response to pulmonary TB, we aerosol inhalated 10 to 14 week-old female 129/Sv wild-type and *Clec7a*^-/-^ mice (generated on a pure 129/Sv background [14]) with ±100 CFU of *Mycobacterium tuberculosis* H37Rv using an inhalation exposure system (Glas-Col, Terre Haute, IN, model no:099 A4212). The infective dose was verified by determining the bacterial loads in the lungs of test mice 24 h after infection, and the remaining animals were subsequently characterised at 2 and 4 months post-infection.

We first examined the pulmonary bacilli burdens, by weighing and homogenizing all the lobes of the right lung (i.e. anterior, posterior middle and post-caval lobes) in saline containing 0.04% Tween 80, and plating serial dilutions of the homogenates onto Middlebrook 7H10 agar enriched with 10% OADC, incubating at 37 °C and counting colonies after 21 days. We found that the bacilli burdens had increased logarithmically from the initial infecting dose and had plateaued, as expected (Fig. 1A) [2]. In comparison to the wild-type animals, however, we observed a significant and reproducible reduction in the CFU in the lungs of the Dectin-1 deficient mice at both time points. Thus the presence of Dectin-1 appeared to contribute to disease susceptibility.

We also explored the effect of Dectin-1 deficiency on pulmonary pathology. Histological sections of the left lung lobes, stained with haemotoxylin and eosin (H&E), from both the wild-type and Dectin-1^−/−^ mice showed similar histological features of pneumonia, consisting of bronchocentric inflammatory infiltrates composed predominantly of lymphocytes and macrophages (Fig. 1B and data not shown). These inflammatory infiltrates extensively involved the alveolated lung tissue, and the alveoli contained numerous foamy macrophages (data not shown). Furthermore, automated morphometric analysis of four serial sections per lung, using a Nikon microscope eclipse 90i and NIS-Elements BR 3.1 9 (Nikon), revealed no difference in the size of the inflammatory lesions in the lungs of wild-type and Dectin-1^−/−^ animals, at both time points (Fig. 1C). These results therefore suggest that the differences in the bacterial burdens do not result in gross histological changes in the lung.

To attempt to determine the underlying reasons for the differences in bacterial burdens, we next examined the expression of pulmonary cytokines in lung homogenates by ELISA (BD Biosciences and R&D Systems), focussing specifically on those cytokines that have previously been shown to be influenced by Dectin-1, including TNF, IL-12, IL-6, IL-17 and IL-10 [8,15]. Although a small increase in the levels of TNF in the Dectin-1^−/−^ mice at 2 months was detected, this increase was not observed at 4 months (Fig. 1D). There were no significant differences between wild-type and Dectin-1 deficient animals in the production of any of the other cytokines tested (Fig. 1D). These data therefore suggest that there are no major changes in the ability of the Dectin-1 deficient animals to induce the production of cytokines during infection with Mtb.

Finally, to explore the effect of Dectin-1 deficiency on survival of mice during pulmonary tuberculosis, we infected wild-type and Dectin-1 deficient mice with 100 CFU of *Mycobacterium tuberculosis* H37Rv and monitored survival of the animals over a period of 150 days (Fig. 1E). Mice displaying ≥20% weight loss were considered moribund and killed, and about 50% of all mice succumbed during the course of the experiment, but we observed no significant alterations in the rate of mortality between wild-type and Dectin-1 deficient animals. This suggests that Dectin-1 does not influence long term survival during infection with *M. tuberculosis*.

Previous studies have suggested that Dectin-1 may play a protective role in anti-mycobacterial immunity, as this receptor has been shown to contribute to the induction of protective inflammatory cytokines, including IL-12, by macrophages and dendritic cells *in vitro* [11–13]. Surprisingly, our results suggest that Dectin-1 may contribute to disease susceptibility, as Dectin-1 deficient mice had reproducibly lower bacilli burdens, when compared to wild-type animals. On the other hand, these reduced bacterial burdens did not correlate with substantial changes in pathology, cytokine production or ability to resist infection.

Our data does not provide conclusive answers to the cause of the reduced bacterial burdens observed in the Dectin-1 deficient mice, but a contributing factor could be alterations in the production of cytokines, particularly TNF and IL-10. Although not significant, there was a tendency towards lower IL-10 levels at both 2 and 4 months. Indeed, in two of the three experiments (which are all pooled in Fig. 1D), we observed significantly less IL-10 at 2 months (*p* = 0.027 and *p* = 0.036, data not shown) in the lungs of the Dectin-1 deficient animals. Given that Dectin-1 can induce IL-10 [8], and the importance of IL-10 in controlling pathogen clearance [3,5], the reductions in the levels of this cytokine may be a factor contributing to the reduced bacterial burdens seen in the Dectin-1 deficient animals. Similarly, the higher levels of TNF (at least at 2 months) may also contribute to Mtb clearance, but in this case it is unclear how Dectin-1 deficiency could lead to such increases, given that this receptor normally stimulates the production of this cytokine [8].

Signalling from Dectin-1 using purified β-glucan as an adjuvant has been shown to drive the development of Th1 and Th17 adaptive immunity, raising the possibility that this receptor is involved in the development of these responses during Mtb infection. [16]. However, we observed no differences in the levels of pulmonary IFNγ or IL-17 in the knockout mice at both 2 or 4 months, suggesting that the development of adaptive immunity is unaffected by Dectin-1 deficiency. Similar results were also observed in these mice during infection with *Candida albicans*, where the main receptor driving adaptive responses was found to be Dectin-2 [17,18]. Indeed, Mincle has recently been shown to be capable of promoting Th1 and Th17 responses to Mtb, and may therefore be the primary receptor promoting the development of adaptive immunity during infection [6,7]

In summary, we have addressed the role of Dectin-1 in the control of Mtb *in vivo*, and conclude that although Dectin-1 contributes to susceptibility, this receptor plays only a minor role in anti-mycobacterial immunity. Determining the mycobacterial ligand recognised by Dectin-1 and the mechanisms underlying the decreased pulmonary bacterial burdens in the receptor deficient mice are issues which need to be addressed in the future.

## Figures and Tables

**Fig. 1:**
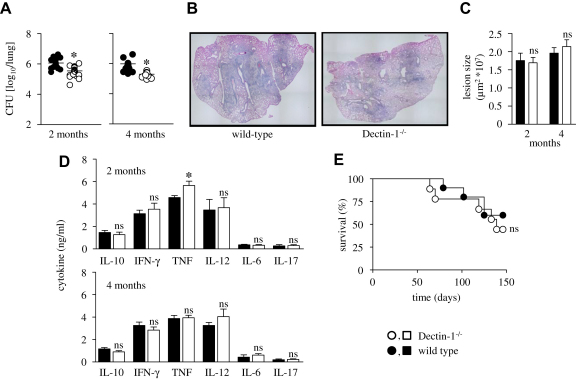
Dectin-1 deficiency leads to a reduction in pulmonary bacilli loads, but no significant changes in pulmonary pathology, cytokine levels or ability of these animals to survive infection with *Mycobacterium tuberculosis* (Mtb). Wild-type (black symbols) and Dectin-1^−/−^ (white symbols) mice (*n* = 4–8 animals per group) were infected with 100 CFU of Mtb H37Rv and at 2 and 4 months post-infection, lungs were analyzed for bacterial burdens (*A*), histopathology (H&E staining at 2 months, *B*), lesion size (2 months, *C*) and selected cytokines (*D*), as indicated. Shown are data pooled from at least two independent experiments and indicate the results from individual animals (*A*), or mean ± SEM (*C*, *D*). Differences between the means of experimental and control group were analyzed with two-tailed student’s *t*-test. (*E*) Wild-type and Dectin-1^−/−^ mice (*n* = 10 per group) were infected with 100 CFU of Mtb and survival was monitored for 150 days. ∗, *p* < 0.05; ns, not significant. Survival data were analyzed with the log rank test.
